# An efficient micropropagation protocol for an endangered ornamental tree species (*Magnolia sirindhorniae* Noot. & Chalermglin) and assessment of genetic uniformity through DNA markers

**DOI:** 10.1038/s41598-019-46050-w

**Published:** 2019-07-03

**Authors:** Yuanyuan Cui, Yanwen Deng, Keyuan Zheng, Xiaomin Hu, Mulan Zhu, Xiaomei Deng, Ruchun Xi

**Affiliations:** 1 Guangdong Key Laboratory for Innovative Development and Utilization of Forest Plant Germplasm, Guangzhou, 510642 China; 2 College of Forestry and Landscape Architecture, South China Agricultural University, Guangzhou, 510642 China; 3 Shanghai Chenshan Plant Science Research Center, Chinese Academy of Sciences, Shanghai, 200000 China; 4 Department of Fruit Tree Sciences, College of Horticulture, China Agricultural University, Beijing, 100193 China

**Keywords:** Plant breeding, Plant physiology

## Abstract

*Magnolia sirindhorniae* Noot. & Chalermglin is an endangered species with high ornamental and commercial value that needs to be urgently protected and judiciously commercialized. In this study, a protocol for efficient regeneration of this species is standardized. The lateral buds of the *M. sirindhorniae* plant were used as an explant. Half-strength Murashige and Skoog (MS) medium supplemented with 2.0 mg/L 6-benzyladenine (BA), 0.1 mg/L α-naphthaleneacetic acid (NAA), and 2.0 mg/L gibberellic acid (GA_3_) was found to be the optimal medium for shoot induction. The maximum shoot multiplication rate (310%) was obtained on Douglas-fir cotyledon revised medium (DCR) fortified with 0.2 mg/L BA, 0.01 mg/L NAA, and additives. The half-strength DCR medium supplemented with 0.5 mg/L NAA and 0.5 mg/L indole-3-butyric acid (IBA) supported the maximum rate (85.0%) of *in vitro* root induction. After a simple acclimatization process, the survival rate of plantlets in a substrate mixture of sterile perlite and peat soil (1:3; *v/v*) was 90.2%. DNA markers were used for assessment of genetic uniformity, confirming the genetic uniformity and stability of regenerated plants of *M. sirindhorniae*. Thus, the described protocol can safely be applied for large scale propagation of this imperative plant.

## Introduction

Magnolias (Magnoliaceae) have long been popular and widely cultivated as ornamental plants, shrubs, and trees. The majority of magnolias are an evergreen species of tree with a graceful form and abundant blooms, typically grown in gardens. Furthermore, many magnolia species have been used in traditional medicine for centuries and many of them have great economic importance as natural sources of aroma and bioactive compounds^1–3^. *Magnolia sirindhorniae* Noot. & Chalermglin, discovered in a freshwater bog in Thailand in 2002, is a new species used in landscaping due to its fast growth, dense foliage, beautiful canopy, and fragrant flowers^4^. Moreover, *M. sirindhorniae* has a unique waterlogging resistance, which makes it perfect and promising for the regreening of the wetland parks. Like other magnolia plants, essential oils can be derived from the leaves and flowers of *M. sirindhorniae*^5,6^. However, due to the decline of its habitat, it was classified as ‘Endangered’ on the IUCN Red List^7^. In addition, it is difficult for *M. sirindhorniae* to reproduce by seeds due to the low percentage of fruit setting.

Plant tissue culture has made a significant contribution to the mass clonal propagation of ornamental and forest trees, providing a large number of superior clonal seedlings in a short time throughout the year^8^. Direct multiple shoot induction is the useful means of producing plantlets from young or mature trees with a lower risk of genetic instability than by the other regeneration routes, and it is a more reliable method for clonal propagation^9,10^. Many endangered magnolia species with high ornamental or commercial value, such as *M. dealbata* and *M. punduana*, have been protected and expanded by *in vitro* propagation^11–13^. However, only one root initiation study of *M. sirindhorniae* has been conducted in which root initiation (90%) was obtained on USK II medium (described by Chaidaroon, 2004) with 4.0 mg/L indole-3-butyric acid (IBA) after 24 days^14^. To date, no studies have reported a protocol for efficient regeneration of this important rare species. Therefore, we explored an efficient protocol for *in vitro* plant regeneration via shoot induction of *M. sirindhorniae* for the mass propagation of this precious magnolia plant.

Under the long-term *in vitro* process, various factors such as media composition and plant growth regulators may result in variations in regenerated plants^15^. Therefore, the genetic uniformity assessment of regenerated plants is of great importance. In recent years, random amplified polymorphic DNA (RAPD) and inter simple sequence repeat (ISSR) have successfully used for assessing the genetic fidelity of regenerated plantlets in many plant species^16–18^.

In the present study, the semi-lignified nodal segments of a 10-year-old *M. sirindhorniae* plant were used as the explants, followed by optimization of protocol for axillary bud induction, cluster bud proliferation, rooting, and acclimatization. The genetic uniformity of regenerated plants was assessed by RAPD and ISSR markers. This research will be of great help to preserve this important species.

## Methods

### Plant material and preparation of explants

**Figure 1:**
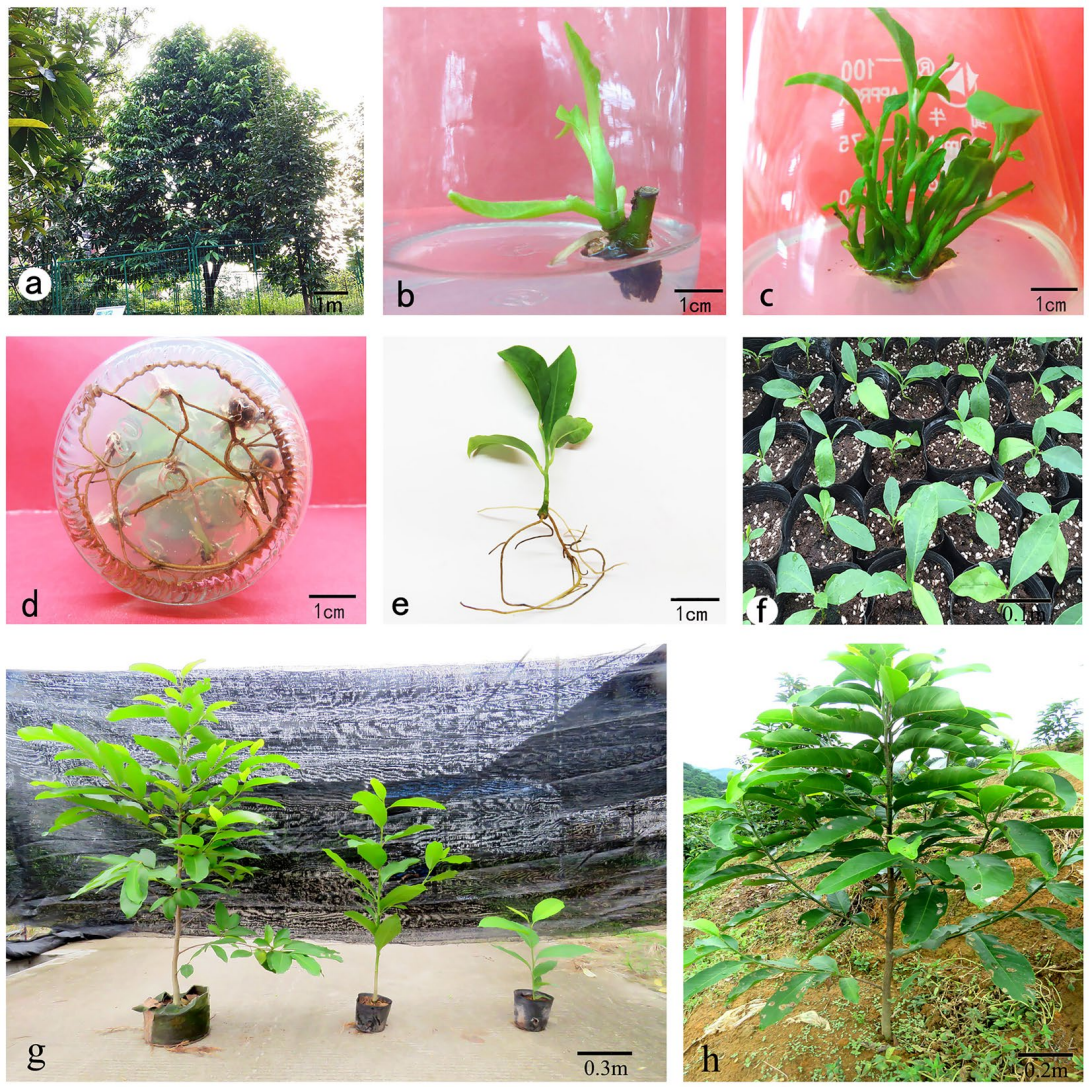
*In vitro* propagation of *M. sirindhorniae* using mature axillary node explants. (**a**) Mature tree; (**b**) Shoot bud initiation. (**c**) Multiple shoot bud regeneration. (**d,e**) Regenerated plantlets with well- developed roots. (**f,h**) Acclimatized plants.

The semi-lignified nodal segments were collected from Shen Zhou Magnolia Park (113°18′E, 23°06′N) in the South China Agricultural University (Fig. 1a). After cleaning in a solution of 5% (*v/v*) liquid detergent, they were washed under running tap water for 1 hour. Further, they were cut into segments (2–5 cm) with one or two buds, then surface-sterilized with 75% (*v/v*) ethanol for 30 s, followed with 0.1% (*w/v*) mercuric chloride solution for 8–20 min. Afterwards, they were rinsed with sterile distilled water five times. After cutting off two ends, the sterilized explants were inoculated vertically on half-strength MS medium supplemented with different concentrations and combinations of plant growth regulators for shoot induction.

### Culture media and growth conditions

Murashige and Skoog (MS)^19^ medium, woody-plant medium (WPM)^20^, Gamborg’s B-5 Basal Medium (B5)^21^ and Douglas-fir cotyledon revised medium (DCR)^22^ were used in this study. All the media were adjusted to pH 5.8, solidified with 7 g/L agar, and autoclaved at 121 °C for 15–18 min. The medium used for the shoot bud induction and multiplication contained 30 g/L sucrose and that used for the rooting contained 15 g/L sucrose. The cultures were incubated in the laboratory at 24 ± 2 °С under a 16/8-h (light/dark cycle) photoperiod provided with cool white fluorescent light (1500–3000 Lx)^23,24^.

### Shoot bud initiation

Half-strength MS medium was chosen as the basal culture medium for shoot induction^25,26^. The effect of different concentrations of diverse plant growth regulators added to half-strength MS medium was compared. Orthogonal design was adopted and repeated three times; each treatment consisted of 10 explants. The concentrations were as follows: 6-benzyladenine (BA): 0.5, 1.0, 2.0 mg/L; α-naphthaleneacetic acid (NAA): 0, 0.05, 0.1 mg/L; gibberellic acid (GA_3_):0, 1.0, 2.0 mg/L. Three levels of each of the three factors were examined in nine experimental runs (orthogonal array L_9_(3^4^))^27,28^. After four weeks of incubation, the percentage of shoot induction, time taken for bud initiation (marked by separation layer on the edges of the petiole), and the growth state of the buds were recorded.

### Shoot proliferation

Nodal segments (1–2 cm) were cut off and transferred into fresh half-strength MS medium supplemented with different concentrations of BA (0.1, 0.2, 0.4, and 0.6 mg/L) in combination with NAA (0.01, 0.02, 0.04, and 0.06 mg/L) individually in order to standardize the maximum rate of shoot multiplication; there were 16 treatments in total. In addition, six different basal culture media (MS, 1/2 MS, 3/4 MS, WPM, B5, and DCR) with the same plant growth regulators were compared during the phase of subculture, and the optimal medium was selected. After four weeks of incubation, the multiplication rate and number of new shoots per explant (≧0.5 cm) were recorded.

### Rooting

Individual shoots (≧1.2 cm height) were cut off and transferred into rooting media. Observations were recorded after every two days^29,30^. To optimize the best root induction medium, half-strength DCR medium was chosen as the rooting medium and was supplemented with different compositions and concentrations of the plant growth regulator: NAA (0.1–1.0 mg/L), IBA (0.1–1.0 mg/L), and cycocel CCC (0.1 and 0.5 mg/L. The percentage of root induction, root numbers, and the growth state of roots were observed and recorded after four weeks.

### Acclimatization

Plantlets that were observed to have well-developed roots after four weeks were transferred to a greenhouse and kept for approximately 5–7 days. Afterwards, the plantlets were gently removed from the culture vessels and washed off the adhering medium. Subsequently, they were transplanted to plastic cups containing mixture of perlite and peat soil in a ratio of 1:3 (*v/v*), which had been disinfected with potassium permanganate solution (1000–1250 ppm). The survival rate was calculated after one month.

### Assessment of genetic uniformity

For genetic fidelity studies, total genomic DNA was extracted from fresh leaves of 18 randomly selected acclimatized plants and their mother plant using the Cetyltrimethyl Ammonium Bromide (CTAB) method^31^. In addition, the total genomic DNA of another *M. sirindhorniae* plant developed from seed was also extracted as the negative control. The concentration of DNA was measured using a NanoDrop 2000 (Thermo Fischer Scientific, Waltham, MA, USA). For RAPD analysis, a total of 18 primers (TsingKe Biological Technology, Tianjin, China) were used according to previous reports^32,33^ and initial experiments. The ISSR analysis was performed with three ISSR primers (TsingKe Biological Technology, Tianjin, China), which had been selected for genetic analysis of Magnolia in previous reports^34,35^.

DNA amplification for RAPD and ISSR markers was performed in a volume of 25 μL reaction mixture containing 2.0 μL of template DNA (50–60 ng), 12.5 μL of 2 × Taq Plus MasterMix (Beijing ComWin Biotech Co., Ltd., Beijing, China), 1.0 μL of 10 μM forward and reverse primer, and 8.5 μL ddH_2_O. RAPD amplification was performed in a thermal cycler (Bio-Rad, Hercules, CA, USA) programmed for initial denaturation at 94 °C for 5 min, followed by 40 cycles of denaturation at 94 °C for 45 s, annealing at 37 for 45 s, and extension at 72 °C for 90 s with a final extension at 72 °C for 10 min. ISSR amplification was performed in a thermal cycler (Bio-Rad) programmed for initial denaturation at 94 °C for 3 min, followed by 35 cycles of denaturation at 94 °C for 30 s, annealing at 54 for 30 s, and extension at 72 °C for 30 s with a final extension at 72 °C for 3 min. All the PCRs were repeated three times, using the same conditions to check the accuracy of the amplified products. Amplified products were electrophoresed in 1.5% agarose gel containing 0.25 μg/mL ethidium bromide (Invitrogen, Carlsbad, CA, USA) using 1x TAE (Tris Acetate EDTA) buffer. The size of the amplification products was estimated by 100 DNA ladder or 5000 bp DNA marker (Takara, Kyoto, Japan). The gels were photographed using the gel documentation system (Bio-Rad, Hercules, CA, USA), only clear and scorable DNA bands were considered.

### Statistical analysis

Induction rate (%) = the number of induced explants/the number of total initial explants × 100%;

Multiplication rate (%) = the total number of buds (≧0.3 cm)/the number of initial buds on the subcultured explants × 100%;

Rooting rate (%) = the number of the rooted plantlets/the number of total shoots × 100%;

Average root numbers = the total number of roots/the number of rooted seedlings.

Each treatment consisted of 10 glass vessels with 4 plantlets, repeated in triplicate. SPSS software version 19.0 was used for the statistical analyses. The significance of differences among means was carried out using Duncan’s multiple range test at P ≤ 0.05 and P ≤ 0.01; The results were represented as mean ± standard error of three replicates.

## Results and Discussion

### Shoot bud induction

The sterilized explants were inoculated into the shoot induction media and then initiated growth after 5–14 days, while petioles began to fall off and small green buds appeared (Table 1a). The axillary bud induction phase was observed between 10–20 days. The higher the concentration of BA added, the earlier the buds sprouted. The range analysis shows that BA had the most influence on the induction rate (Table 1b). The induction rate was only 68% in the case of low BA concentration and the lateral buds initiated late; additionally, the new buds were thin and delicate. The advantageous BA concentration is 2.0 mg/L, and the induction rate reached 79%. Previous studies also showed that BA induced the maximum response. The superiority of BA over other cytokinins was reported by Hashem, Bekircan and Hussain^36–38^. GA_3_ contributes to the initiation and elongation of axillary buds as well as leaf expansion; the axillary buds began to grow after five days when GA_3_ was added. Comprehensively considering growth and induction rate, half-strength MS medium supplemented with 2.0 mg/L BA, 0.1 mg/L NAA, and 1.0 mg/L GA_3_ turned out to be a better medium for *in vitro* induction, as it supported maximum shoot bud induction (Fig. 1b).

**Table 1:** Effect of bud induction by different compositions and concentrations of plant growth regulators and range analysis.

Test number	BA mg/L	NAA mg/L	GA_3_ mg/L	Induction rate % (mean ± SE)	Time of initiation	Growth state of buds
**a**						
1	0.5	0	0	37.06 ± 0.86^Dd^	14th day	+
2	0.5	0.05	1.0	61.37 ± 1.78^BCbc^	10th day	++
3	0.5	0.1	2.0	59.10 ± 1.22^Cc^	10th day	++
4	1.0	0	1.0	68.03 ± 0.69^ABCabc^	8th day	+++
5	1.0	0.05	2.0	67.43 ± 2.01^ABCabc^	8th day	+++
6	1.0	0.1	0	76.13 ± 1.56^ABa^	10th day	+++
7	2.0	0	2.0	75.23 ± 0.69^ABa^	5th day	++++
8	2.0	0.05	0	72.00 ± 1.36^ABabc^	7th day	++++
9	2.0	0.1	1.0	79.03 ± 2.75^Aa^	7th day	++++
**b**						
Range Analysis						
K_BA0.5_	52.533	K_NAA0_	60.100	K_GA0_	61.733	
K_BA1.0_	70.500	K_NAA0.05_	66.933	K_GA1.0_	69.467	
K_BA2.0_	75.400	K_NAA0.1_	71.400	K_GA2.0_	67.233	
R_BA_	22.867	R_NAA_	11.300	R_GA_	7.734	

++++: vigorous and green buds; +++: healthy buds; ++: weak buds; +: unhealthy buds.

Each value represents the mean ± SE of three replicates. Different lowercase letters in the same column indicated the significant difference at P ≤ 0.05; Different uppercase letters in the same column indicated the significant difference at P ≤ 0.01. (Duncan’s multiple range test).

Kxy means sum of induction rate at the y level of x; R (Range) represents measures of variation, Rx=Kxmax-Kxmin.

GA_3_ has been shown to modulate the growth and development of plants, mainly by stimulating mitotic division and cell elongation^39,40^. The positive effects of GA_3_ on bud break have been reported in the tissue culture of woody plant species^41–44^. However, adding the improper concentration of GA_3_ has a negative effect. It was found that a high level of GA_3_ effectively increased shoot length, whereas a lower concentration of GA_3_ inhibited shoot growth in *in vitro* culture of potato^45^. GA_3_ has been used to break dormancy and stimulate shoot elongation in different species of magnolias for a long time^46–48^. In the present study, GA_3_ was found to be important for bud induction, effectively shortening the time of initiation and inducing stronger buds, which is consistent with previous reports^49,50^. However, some plants cultured *in vitro* did not undergo any significant growth stimulation with GA_3_ ^51^. Furthermore, GA_3_ at any concentration induced the formation of malformed plants in *in vitro* culture of *Annona emarginata*^52^. Therefore, GA_3_ should be used conservatively in tissue culture. Different species have different responses to GA_3_, and even different genotypes of the same species have different responses to GA_3_.

### Shoot bud proliferation

The basal medium is an important substrate for plant tissue culture. Due to the genetic, biological and ecological characteristics of various plants, the nutritional components required by various plants are not the same, and the requirements for the composition of the medium are also different. Therefore, choosing the right type of medium is crucial for the success of plant tissue culture^53,54^. Shoot buds from explants were subcultured on six media supplemented with 0.2 mg/L BA in combination with 0.01 mg/L NAA to screen the optimal medium. Among the six tested media (Table 2), the best shoot bud proliferation and elongation were observed on DCR medium, which proliferated to 2.92 times than the original after four weeks (Fig. 1c). Although there was no significant difference observed in the multiplication rate and shoot numbers between MS, 1/2MS, WPM, and DCR media, the growth state of the buds was totally different. The bud clusters on the DCR medium were verdant green and thriving, showing no defoliation, vitrification, or callus.

**Table 2:** Effect of different basal media on bud proliferation.

Basal media	Multiplication rate % (mean ± SE)	Shoot numbers per explant (≧0.5 cm) (mean ± SE)	Growth state of buds	Description
MS	294.33 ± 6.12^a^	1.48 ± 0.30^abc^	++	Hyperhydricity, callus
1/2MS	275.00 ± 105.52^ab^	1.83 ± 0.58^a^	++	Crinkle leaf, callus
3/4MS	238.00 ± 24.22^bc^	1.39 ± 0.47^bc^	+	Hyperhydricity, callus
DCR	292.33 ± 13.78^a^	1.85 ± 0.21^a^	++++	
WPM	275.00 ± 23.28^ab^	1.63 ± 0.93^ab^	+++	
B5	204.67 ± 8.65^c^	1.11 ± 0.23^c^	+	Flavescent, defoliation

++++: vigorous and green buds; +++: healthy buds; ++: weak buds; +: unhealthy buds.

Each value represents the mean ± SE of three replicates. Different lowercase letters in the same column indicated the significant difference at P ≤ 0.05. (Duncan’s multiple range test).

Further, shoot buds were subcultured on DCR medium supplemented with different combinations of BA and NAA to screen for the optimal combination and concentration of BA and NAA (Table 3). Apparently, there was an increase in the multiplication rate with the increase of BA concentration under the same auxin level. On the contrary, it decreased both in the multiplication rate and shoot numbers with increasing NAA concentration under the same cytokinin level. Higher number of multiple shoots occurred on the media with high BA concentrations. Defoliation and vitrification occurred when NAA concentration reached 0.06 mg/L. It is known that plantlets do not grow well when the level of growth regulator is high. Cytokinin promoted the optimal proliferation at low concentrations Among the various combinations tested, the highest rate (299%) of multiplication was observed on the medium fortified with the combination of 0.6 mg/L BA and 0.01 mg/L NAA. However, the optimal growth state of the buds as well as the shoot length (≧0.5 cm) was found on the medium fortified with the combination of 0.2 mg/L BA and 0.01 or 0.02 mg/L NAA (Table 3). In conclusion, the latter two combinations were more suitable for shoot bud proliferation and elongation (Fig. 1c).

**Table 3:** Effect of different compositions and concentrations of BA and NAA on bud proliferation.

BA mg/L	NAA mg/L	Multiplication rate % (mean ± SE)	Shoot numbers per container (≧0.5 cm) (mean ± SE)	Growth state of buds	Description
0.1	0.01	274.00 ± 11.00^ABCbcde^	5.25 ± 0.50^BCDbc^	+	Small buds
0.1	0.02	270.33 ± 15.95^ABCDcde^	4.51 ± 0.25^Dc^	+	
0.1	0.04	244.00 ± 9.85^DEfg^	4.48 ± 0.75^Dc^	+	Defoliation
0.1	0.06	228.67 ± 21.57^Eg^	4.75 ± 0.75^CDc^	++	Defoliation
0.2	0.01	281.00 ± 6.25^ABCabcd^	7.00 ± 0.25^Aa^	++++	
0.2	0.02	296.67 ± 5.13^ABab^	7.00 ± 0.50^Aa^	++++	
0.2	0.04	266.33 ± 7.51^BCDde^	6.08 ± 0.38^ABCab^	+++	
0.2	0.06	235.33 ± 15.04^Eg^	4.83 ± 0.52^CDc^	+++	Defoliation
0.4	0.01	290.33 ± 6.03^ABabc^	6.63 ± 0.25^ABa^	++	Crinkle leaf
0.4	0.02	275.33 ± 10.60^ABCbcde^	5.33 ± 0.54^BCDbc^	++	Crinkle leaf
0.4	0.04	236.00 ± 20.95^Eg^	5.18 ± 0.86^CDc^	++	
0.4	0.06	228.67 ± 6.81^Eg^	4.83 ± 0.90^CDc^	++	Hyperhydricity
0.6	0.01	299.00 ± 13.12^Aa^	6.75 ± 0.50^ABa^	+++	Dense buds
0.6	0.02	293.00 ± 6.25^ABabc^	6.50 ± 0.50^ABCbcde^	++	Dense buds
0.6	0.04	280.33 ± 11.93^ABCabcd^	5.25 ± 0.50^BCDbc^	+	Flavescent
0.6	0.06	257.67 ± 7.23^CDEef^	4.67 ± 0.89^CDc^	+	Hyperhydricity

++++: vigorous and green buds; +++: healthy buds; ++: weak buds; +: unhealthy buds.

Each value represents the mean ± SE of three replicates. Different lowercase letters in the same column indicated the significant difference at P ≤ 0.05; Different uppercase letters in the same column indicated the significant difference at P ≤ 0.01. (Duncan’s multiple range test).

Hyperhydricity is a physiological malformation that results in excessive hydration, yellowing, swelling, glassiness, and leaf curling, which directly affects propagation^55^. In the present study, *M. sirindhorniae* plantlets that were grown showed signs of being hyperhydrated when cultured on MS medium and 3/4 MS medium that contained high concentrations of nitrate, especially with higher BA (Tables 2 and 3). Hyperhydricity disappeared when the medium was changed and the concentration of plant growth regulators was reduced. It was reported that hyperhydricity was positively correlated with tissue nitrate content and cytokinin concentration^56,57^, which may explain why plantlets are hyperhydrated on MS medium or 3/4 MS medium. However, it is unlikely that the tissue nitrate level alone directly affects hyperhydricity. It was also reported that ventilation of culture vessels and using the proper gelling agent can relieve hyperhydricity; the use of gelrite resulted in almost four times higher hyperhydricity compared to agar-solidified medium^58–60^. Using a ventilated culture vessel was proven to be useful to relieve hyperhydricity for *in vitro* plantlets of *M. sirindhorniae*.

### Rooting

The inability to induce adventitious roots is often a limiting factor in conventional cuttings and tissue culture. In an earlier review, the plants in Magnoliaceae had difficulty with root formation^61,62^. It was reported that *in vitro* Magnoliaceae shoots had difficulty with rhizogenesis under the low concentration of plant growth regulators and only produced a large amount of calluses^63^. Therefore, CCC was specifically added to the rooting media for the purpose of reducing the generation of calluses^29^. However, the supplementation of the medium with CCC did not result in successful rooting. The maximum percentage of rooting (95.67%) with plentiful lateral roots and slight callus as well as the highest average root number of 1.87 was observed on half-strength DCR medium supplemented with 0.5 mg/L NAA and 0.5 mg/L IBA (Table 4, Fig. 1d,e). This rooting medium is more efficient compared with that used in previous research^14^. The percentage of rooting first increased and then declined with the increasing concentration of auxins, which was consistent with the *in vitro* rooting studies of other woody plants^12,13,64^. Besides, the quality of the subculture shoots evidently influenced rooting. As a consequence, it is important to obtain healthy normal shoots in the phase of multiplication culture.

**Table 4:** Effects of auxins on the rooting of *M. sirindhorniae*.

Plant growth regulators combination mg/L	Percentage of rooting %	Root numbers (mean ± SE)	Description
NAA 0.5 + IBA 1	54.33 ± 13.90^Bc^	1.20 ± 0.20^ABbc^	Callus
NAA 1 + IBA 1 + CCC 0.1	85.33 ± 6.10^ABab^	1.45 ± 0.24^ABabc^	Callus, no lateral roots
NAA 0.5 + IBA 1 + CCC 0.5	85.00 ± 6.10^ABab^	1.57 ± 0.19^ABab^	Callus
NAA 1 + CCC 0.1	75.33 ± 11.20^ABabc^	1.67 ± 0.21^ABab^	Plenty of callus
NAA 1 + CCC 0.5	70.67 ± 8.90^ABabc^	1.40 ± 0.16^ABabc^	Plenty of callus
NAA 1	60.00 ± 12.70^ABbc^	1.70 ± 0.13^ABab^	Plenty of callus
NAA 0.5 + IBA 0.5	95.67 ± 5.00^Aa^	1.87 ± 0.18^Aa^	Slight callus, plentiful lateral roots
IBA 1	55.50 ± 7.30^Bc^	1.67 ± 0.21^ABab^	Callus, no lateral roots
NAA 0.1 + IBA 0.1	1.60 ± 10.20^Cd^	1.00 ± 0.45^Bc^	Plenty of callus
0	1.60 ± 8.40^Cd^	1.00 ± 0.38^Bc^	

Each value represents the mean ± SE of three replicates. Different lowercase letters in the same column indicated the significant difference at P ≤ 0.05; Different uppercase letters in the same column indicated the significant difference at P ≤ 0.01. (Duncan’s multiple range test).

### Acclimatization

The acclimatization of tissue cultured plants was the most difficult and labor-consuming step because the newly transplanted plantlets were highly susceptible to fungal diseases^65^. In the present study, the rooted plantlets were successfully transferred into plastic cups containing a perlite and peat soil mixture at a ratio of 1:3 followed by a series of effective protection measures. After being transplanted, the plantlets must be watered and then covered with plastic film and shading net to maintain high humidity. Additionally, it is necessary to spray the plantlets with a carbendazim solution to increase plant tolerance to environmental stresses. Ventilation and removal of fallen leaves and rotten seedling should be performed in a timely manner to prevent plant diseases and insect pests. The plastic film and shading net was removed after two weeks. The survival rate of plantlets reached 90.2% (Fig. 1f,g). After lignification, they were transferred to the field. Regenerated plants grew well in the field and were phenotypically similar to the mother stock (Fig. 1h).

### Assessment of genetic uniformity of regenerated plants

**Figure 2:**
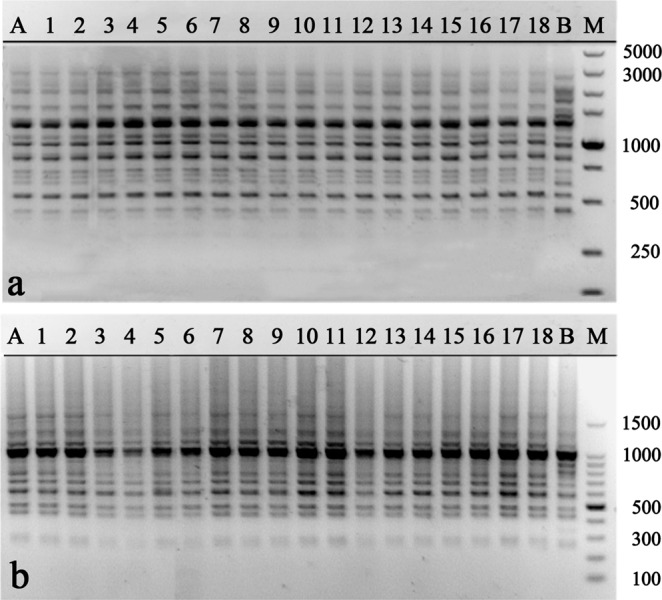
RAPD profiles generated by PCR amplification with primer S10 (**a**), S30 (**b**). Lane M: Molecular marker (100 bp–5 Kb for S10; 100 bp–1.5 Kb for S30); Lane A: Mother plant; Lane 1–18: Regenerated plants; Lane B: Another *M. sirindhorniae* plant developed from seed (negative control).

**Figure 3:**
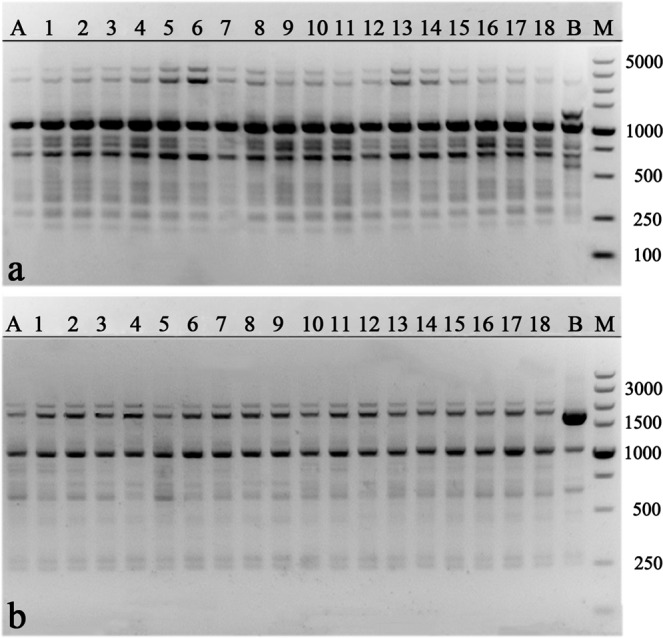
ISSR profiles generated by PCR amplification with primer UBC842 (**a**), UBC855 (**b**). Lane M: Molecular marker (100 bp–5 Kb); Lane A: Mother plant; Lane 1–18: Regenerated plants; Lane B: Another *M. sirindhorniae* plant developed from seed (negative control).

Compared with the natural environment, *in vitro* culture is more complicated and stressful, which is more likely to cause genetic variation^66^. Therefore, it is necessary to assess the genetic uniformity of the regenerated plants before confirming the success of a micropropagation protocol. In the present study, a total of 174 bands were generated by RAPD and ISSR markers with an average of 8.3 bands per primer (Table 5). Eighteen RAPD primers generated 152 clear and scorable bands in total, ranging from 250 to 4000 bp. The number of bands generated by a single RAPD primer varied from 5 to 13 (Table 5, Fig. 2). Three ISSR primers generated 22 clear and scorable bands in total, ranging from 250 to 5000 bp. The number of bands generated by a single ISSR primer varied from 6 to 9. Compared with the negative control, no polymorphic bands were detected between mother plant and regenerated plants, confirming the genetic uniformity and stability of regenerated plants of *M. sirindhorniae* (Table 5, Fig. 3). Our results demonstrate that axillary shoot proliferation minimizes the chance of instability, consistent with previous reports^17,67,68^. This is the first report of genetically sustainable micropropagation in Magnolia plants.

**Table 5:** List of primers, their sequences, number, and size of the amplified fragments generated by 18 RAPD and 3 ISSR markers.

Primer code	Primer sequence (5′–3′)	No. of scorable bands	Approximate Range of amplification (bp)
**RAPD**			
S10	CTGCTGGGAC	12	250–3,000
S11	GTAGACCCGT	5	500–3,000
S17	AGGGAACGAG	6	250–3,000
S18	CCACAGCAGT	6	500–3,000
S22	TGCCGAGCTG	10	250–2,000
S30	GTGATCGCAG	9	200–2,000
S31	CAATCGCCGT	6	250–3,000
S38	AGGTGACCGT	11	400–2,000
S40	GTTGCGATCC	7	400–1,500
S69	CTCACCGTCC	10	300–1,500
S144	GTGACATGCC	8	500–1,500
S154	TGCGGCTGAG	8	500–1,500
S155	ACGCACAACC	9	600–3,000
S158	GGACTGCAGA	8	400–2,000
S160	AACGGTGACC	13	200–3,000
S163	CAGAAGCCCA	8	300–2,000
S173	CTGGGGCTGA	9	500–2,000
S174	TGACGGCGGT	7	500–4,000
**Total**		**152**	
**ISSR**			
UBC840	(GA)_8_CTT	9	300–1,500
UBC842	(GA)_8_CTG	7	250–5,000
UBC855	(AC)_8_YT	6	250–3,000
**Total**		**21**

## Conclusions

The present report describes an efficient protocol for large-scale micropropagation from axillary nodal explants of *M. sirindhorniae*. Direct multiple shoot induction suppresses the risk of genetic instability. The maximum shoot bud induction (79.0%) occurred on 1/2 MS medium supplemented with 2.0 mg/L BA, 0.1 mg/L NAA, and 2.0 mg/L GA_3_. It turned out that DCR medium was the best basic medium for *in vitro* propagation of *M. sirindhorniae*, and the highest proliferation rate (310%) was obtained on DCR medium fortified with 0.2 mg/L BA and 0.01 mg/L NAA. Half-strength DCR medium supplemented with 0.5 mg/L NAA and 0.5 mg/L IBA was proven to be the best for rooting, and the highest rooting percent (nearly 96%) was achieved in spite of the fact that it is difficult for *Magnoliaceae* plants to root in plant tissue culture. The regenerated plantlets were well-acclimatized to the wild. RAPD and ISSR markers confirmed the genetic uniformity of regenerated plants. Hence, this protocol can be successfully used for the commercial multiplication of *M. sirindhorniae*.

## Data Availability

The datasets generated during and/or analyzed during the current study are available from the corresponding author on reasonable request.

## References

[1] Lee YJ (2011). Therapeutic applications of compounds in the Magnolia family. Pharmacol. Ther..

[2] Farag MA, El Din RS, Fahmy S (2015). Headspace analysis of volatile compounds coupled to chemometrics in leaves from the Magnoliaceae family. Rec. Nat. Prod..

[3] Morshedloo MR, Quassinti L, Bramucci M, Lupidi G, Maggi F (2017). Chemical composition, antioxidant activity and cytotoxicity on tumour cells of the essential oil from flowers of *Magnolia grandiflora* cultivated in Iran. Nat. Prod. Res..

[4] Nooteboom HP, Chalermglin P (2009). The Magnoliaceae of Thailand. Thai Forest Bulletin (Botany)..

[5] Li, J. Essential oil extraction of Magnoliaceae plants and GC - MS analysis. Doctoral dissertation, South China University of Technology (2011).

[6] Katekunlaphan T, Chalermglin R, Rukachaisirikul T, Chalermglin P (2014). Sesquiterpene lactones from the leaves of *Magnolia sirindhorniae*. Biochemical Systematics & Ecology..

[7] Rivers, M., Beech, E., Murphy, L. & Oldfield S. The Red List of Magnoliaceae (Revised and Extended). Botanic Gardens Conservation International, Richmond, UK (2016).

[8] Shahzad, A. *et al*. Plant Tissue Culture: Applications in Plant Improvement and Conservation. In *Plant Biotechnology: Principles and Applications* (pp. 37–72). Springer Singapore (2017).

[9] Giri CC, Shyamkumar B, Anjaneyulu C (2004). Progress in tissue culture, genetic transformation and applications of biotechnology to trees: an overview. Trees..

[10] Jani JN, Jha SK, Nagar DS (2015). Root explant produces multiple shoot from pericycle in *Psoralea corylifolia*–a leprosy destroyer medicinal plant. Ind. Crop. Prod..

[11] Matarosas M, JiméNezrodríGuez A (2006). Somatic embryogenesis and organogenesis in *Magnolia dealbata zucc*. (Magnoliaceae), an endangered, endemic Mexican species. Hortscience A Publication of the American Society for Horticultural Science..

[12] DomãNguez F (2010). Honokiol and magnolol production by *in vitro* micropropagated plants of *Magnolia dealbata*, an endangered endemic Mexican species. Nat. Prod. Commun..

[13] Borah R, Kumaria S, Choudhury H (2017). *In vitro* plant regeneration of *Magnolia punduana*: an endemic and threatened plant species. Plant Tissue Culture & Biotechnology..

[14] Chaidaroon, S., Ungvichian, I., Ratanathavornkiti, K. *In vitro* root initiation of ‘Champi Sirindhorn’ (*Magnolia sirindhorniae* Noot. & Chalermglin). *Assumption Univ. J. Tech. Jan*. 129–132 (2004).

[15] Bennici A, Anzidei M, Vendramin GG (2004). Genetic stability and uniformity of *Foeniculum vulgare* Mill. regenerated plants through organogenesis and somatic embryogenesis. Plant science.

[16] Chavan JJ (2015). Highly efficient *in vitro* proliferation and genetic stability analysis of micropropagated *Ceropegia evansii* by RAPD and ISSR markers: a critically endangered plant of Western Ghats. Plant Biosystems-An International Journal Dealing with all Aspects of Plant Biology.

[17] Ahmed MR, Anis M, Alatar AA, Faisal M (2017). *In vitro* clonal propagation and evaluation of genetic fidelity using RAPD and ISSR marker in micropropagated plants of *Cassia alata* L.: a potential medicinal plant. Agrofor. Syst..

[18] Tikendra, L., Koijam, A. S. & Nongdam, P. Molecular markers based genetic fidelity assessment of micropropagated *Dendrobium chrysotoxum* Lindl. Meta Gene. 100562 (2019).

[19] Murashige T, Skoog F (1962). A revised medium for rapid growth and bio assays with tobacco tissue cultures. Physiol. Plant..

[20] Lloyd G, Mccown B (1980). Commercially-feasible micropropagation of mountain laurel, kalmia latifolia, by use of shoot-tip culture. *Combined Proceedings-International Plant Propagators’*. Society (USA)..

[21] Gamborg OL, Miller R, Ojima K (1968). Nutrient requirements of suspension cultures of soybean root cells. Experimental cell research..

[22] Gupta PK, Durzan DJ (1985). Shoot multiplication from mature trees of Douglas-fir (*Pseudotsuga menziesii*) and sugar pine (*Pinus lambertiana*). Plant Cell Reports..

[23] Ailian Zhang (2015). Large-scale *in vitro* propagation of *Anoectochilus roxburghii* for commercial application: pharmaceutically important and ornamental plant. Ind. Crop. Prod..

[24] Anjusha S, Gangaprasad A (2016). *In vitro* propagation and anthraquinone quantification in *Gynochthodes umbellata* (L.) Razafim. & B. Bremer (Rubiaceae)—A dye yielding plant. Ind. Crop. Prod..

[25] Gan L, Li K, Wang X, Chen X (2010). A preliminary study on the tissue culture of *Parakmeria yunnanensis* (Magnoliaceae). Guangxi Agricultural Sciences..

[26] Wang, Z. Regeneration and total phenol content change research of *Magnolia officinalis*. Doctoral dissertation, South Central Forestry S&T university (2013).

[27] Cheng T, Voqui TH (1977). Regeneration of Douglas fir plantlets through tissue culture. Science.

[28] Zhang Z (1985). Application of Orthogonal Design in Plant Tissue Culture. J. Plant Physiol..

[29] Jieru Z (2014). The influence of ccc in rooting and transplanting of *Hemerocallis fulva*. Journal of Northeast Forestry University..

[30] Cheng Q (2014). Rapid propagation system of *Michelia crassipes*. China Forestry Science and Technology..

[31] Porebski S, Bailey LG, Baum BR (1997). Modification of a CTAB DNA extraction protocol for plants containing high polysaccharide and polyphenol components. Plant Mol. Biol. Rep..

[32] Jiang, J. Genetic resource and evaluation of the Magnoliaceae species and cultivar breeding of *Michelia chapensis* Dandy. Doctoral dissertation, Chinese Academy of Forestry (2006).

[33] Zheng, Z. Studies on genetic diversity and construction of fingerprinting of *Magnolia officinalis*. Doctoral dissertation, Fujian Agriculture and Forestry university (2010).

[34] Huang, L. RAPD and ISSR analysis of 20 species in 6 genera from Magnoliaceae. Doctoral dissertation, Fujian Normal University (2007).

[35] Medrano-Hernández, J. M., Reyes-Trejo, B. & Peña-Ortega, M. G. Molecular characterization using ISSR primers of Magnolia mexicana DC. from two regions in Zongolica, Veracruz, Mexico. Revista Chapingo. *Serie Ciencias Forestales y del Ambiente*, **23**(3) (2017).

[36] Hashem AH, Razzooqee MA, Salim SAAR (2015). Effect of different concentrations of Turmeric (*Curcuma longa* L.) powder and BA on *in vitro* direct organogenesis from cotyledons of mandarin (*Citrus reticulata* Blanco). Journal of kerbala university..

[37] Bekircan T, Yaşar A, Yıldırım S, Sökmen M, Sökmen A (2018). Effect of cytokinins on *in vitro* multiplication, volatiles composition and rosmarinic acid content of *Thymus leucotrichus* Hal. shoots. 3 Biotech..

[38] Hussain SA, Ahmad N, Anis M (2018). Synergetic effect of TDZ and BA on minimizing the post-exposure effects on axillary shoot proliferation and assessment of genetic fidelity in *Rauvolfia tetraphylla* (L.). Rend. Lincei. -Sci. Fis. Nat..

[39] Kumar HK, Chandana E, Chauhan JB (2012). *In vitro* propagation of *Calamus nagbettai*: an endangered plant. J Microbiol Biotechnol Res..

[40] Ali S (2018). *In vitro* effects of GA_3_ on morphogenesis of CIP potato explants and acclimatization of plantlets in field. In Vitro Cell. Dev. Biol.-Plant..

[41] Dimassi-Theriou, K. Effects of exogenous ethylene, CO_2_ and GA_3_ on shoot proliferation *in vitro* of sweet cherry (*Prunus avium* L.). *Advances in horticultural science*. 38–42 (1998).

[42] El-Agamy, S. Z., Mohamed, A. K. A., Mostafa, F. M. A. & Abdallah, A. Y. Effect of GA3, hydrogen cyanamid and decapitation on budbreak and flowering of two apple cultivars under the warm climate of Southern Egypt. *In VI International Symposium on Temperate Fruit Growing in the Tropics and Subtropics 565* **(pp. 109–114)** (2000).

[43] Qin D (2017). Effects of GA3 and ABA on the respiratory pathways during the secondary bud burst in black currants. J. For. Res..

[44] Zheng C (2018). Distinct gibberellin functions during and after grapevine bud dormancy release. J. Exp. Bot..

[45] Sabeti M, Zarghami R, Zadeh ME (2013). Effects of explants and growth regulators on callogenesis and somatic embryogenesis of Agria potato cultivar. International Journal of Agriscience..

[46] Sahoo Y, Chand PK (1998). Micropropagation of *Vitex negundo* L., a woody aromatic medicinal shrub, through high-frequency axillary shoot proliferation. Plant Cell Reports..

[47] Fernando, M. T. R., Jayasuriya, K. G., Walck, J. L. & Wijetunga, A. S. T. B. Identifying dormancy class and storage behaviour of champak (*Magnolia champaca*) seeds, an important tropical timber tree. *J. Natl. Sci. Found. Sri Lanka*. **41**(2) (2013).

[48] Iralu V, Upadhaya K (2016). Dormancy, storability, and germination of seeds in *Magnolia punduana* (Magnoliaceae). Botany..

[49] Sokolov RS, Atanassova BY, Iakimova ET (2014). Physiological response of *in vitro* cultured Magnolia sp. to nutrient medium composition. Journal of Horticultural research..

[50] Wojtania A, Skrzypek E, Gabryszewska E (2016). Morphological and biochemical responses to gibberellic acid in Magnolia × ‘Spectrum’ *in vitro*. Acta Biologica Cracoviensia s. Botanica..

[51] Nagori R, Purohit SD (2004). *In vitro* plantlet regeneration in *Annona squamosa*, through direct shoot bud differentiation on hypocotyl segments. Sci. Hortic..

[52] de Freitas RT (2016). *In vitro* culture of *Annona emarginata*: a rootstock for commercial Annonaceae species. Plant Cell Culture & Micropropagation..

[53] Schenk RU, Hildebrandt AC (1972). Medium and techniques for induction and growth of monocotyledonous and dicotyledonous plant cell cultures. Canadian Journal of Botany..

[54] George, E. F., Hall, M. A. & De Klerk, G. J. Plant tissue culture procedure-background. *In Plant propagation by tissue culture* (pp. 1–28). Springer, Dordrecht (2008).

[55] Kevers C, Franck T, Strasser RJ, Dommes J, Gaspar T (2004). Hyperhydricity of micropropagated shoots: a typically stress-induced change of physiological state. Plant Cell Tissue Organ Cult..

[56] Brand MH (1993). Agar and ammonium nitrate influence hyperhydricity, tissue nitrate and total nitrogen content of serviceberry (*Amelanchier arborea*) shoots *in vitro*. Plant Cell Tissue Organ Cult..

[57] Kadota M, Niimi Y (2003). Effects of cytokinin types and their concentrations on shoot proliferation and hyperhydricity in *in vitro*, pear cultivar shoots. Plant Cell Tissue Organ Cult..

[58] Park SW (2004). Effect of sealed and vented gaseous microenvironments on the hyperhydricity of potato shoots. in vitro. Sci. Hortic..

[59] Lai CC (2005). Hyperhydricity in shoot cultures of *Scrophularia yoshimurae* can be effectively reduced by ventilation of culture vessels. J. Plant Physiol..

[60] Ivanova M, Van Staden J (2011). Influence of gelling agent and cytokinins on the control of hyperhydricity in *Aloe polyphylla*. Plant Cell Tissue Organ Cult..

[61] Ma, L. Y., Huai, H. M. & Jia, Z. K. Callus induction method of *Magnolia wufengensis* (Magnoliaceae). CN 102860255 A (2013).

[62] Gao Z (2013). Research progress of rapid propagation of Magnoliaceae in China. Science of Jiangxi..

[63] Deng X, Xi R, Fu S (2007). Establishment of highly efficient regeneration system of *Parakmeria lotungensis*. Chun et C. Tsoong Law. Acta Agriculturae Universitatis Jiangxiensis..

[64] Ali A, Ahmad T, Abbasi NA, Hafiz IA (2009). Effect of different concentrations of auxins on *in vitro* rooting of olive cultivar ‘Moraiolo’. Pak. J. Bot..

[65] Timofeeva SN, Elkonin LA, Tyrnov VS (2014). Micropropagation of *Laburnum anagyroides*, Medic. through axillary shoot regeneration. In Vitro Cell. Dev. Biol.-Plant..

[66] Bairu MW, Aremu AO, Van Staden J (2011). Somaclonal variation in plants: causes and detection methods. Plant Growth Regul..

[67] Phulwaria M, Rai MK, Shekhawat NS (2013). An improved micropropagation of *Arnebia hispidissima* (Lehm.) DC. and assessment of genetic fidelity of micropropagated plants using DNA-based molecular markers. Appl. Biochem. Biotechnol..

[68] Saha S, Roy S, Sengupta C, Ghosh P (2014). Micropropagation and analysis of genetic stability in regenerated plantlets of *Ocimum canum* Sims. Indian Journal of Plant Physiology..

